# Speed Matters in Nordic Hamstring Exercise: Higher Peak Knee Flexor Force during Fast Stretch-Shortening Variant Compared to Standard Slow Eccentric Execution in Elite Athletes

**DOI:** 10.3390/sports11070130

**Published:** 2023-07-07

**Authors:** Jesper Augustsson, Tobias Alt, Håkan Andersson

**Affiliations:** 1 Department of Sport Science, Faculty of Social Sciences, Linnaeus University, 39182 Kalmar, Sweden; 2 Department of Biomechanics, Performance Analysis and Strength & Conditioning, Olympic Training and Testing Centre Westphalia, 44139 Dortmund, Germany; tobias.alt@osp-westfalen.de; 3 High Performance Center, Strength and Conditioning Institute, 35246 Vaxjo, Sweden; hakan.andersson@hpcsweden.com

**Keywords:** eccentric, concentric, strength training, knee flexor strength, injury prevention

## Abstract

Hamstring strain injuries are prevalent in many sports. Research has demonstrated that the Nordic hamstring exercise (NHE), a knee-dominant exercise addressing the posterior chain muscles, can aid in reducing the risk of hamstring injuries in athletes. However, most research on hamstring injury prevention has focused on performing the eccentric version of the NHE (NHE_ECC_). In contrast, in sports, it is quite frequent for athletes to use an eccentric–concentric version of the NHE. Additionally, eccentric NHE is typically performed using a slow, controlled tempo. The effect of a fast stretch-shortening cycle NHE (NHE_SSC_) compared to standard slow NHE_ECC_ on peak knee flexor force has not been investigated. The aim of the study was therefore to investigate fast NHE_SSC_ vs. standard slow NHE_ECC_. Our hypothesis posited that peak knee flexor force would be greater for fast NHE_SSC_ compared with standard slow NHE_ECC_. The study involved 22 elite athletes (actively competing in both national and international events) consisting of female (*n* = 10) and male (*n* = 7) track and field athletes and male football players (*n* = 5), aged 17–31 years. The participants performed maximum trials of slow NHE_ECC_ and fast NHE_SSC_ repetitions in which measurement of bilateral peak knee flexor force was conducted at the ankle with the use of a load cell. During the NHEs, a linear encoder was used to measure both the position where the peak knee flexor force was recorded and the average eccentric velocity. SSC contributed to an enhanced NHE performance, where bilateral absolute peak knee flexor force was 13% higher for fast NHE_SSC_ vs. standard slow NHE_ECC_ (822 vs. 726 N, *p* < 0.01, ES = 0.54). Participants achieved a 32% greater forward distance at the breakpoint stage during NHE_ECC_ compared to the coupling phase for NHE_SSC_ (54 vs. 41 cm, *p* < 0.001, ES = 1.37). Eccentric average velocity was more than three times higher for NHE_SSC_ compared with NHE_ECC_ (0.38 vs. 0.12 m/s, *p* < 0.001, ES = 3.25). The key findings of this study were that SSC contributed to an enhanced NHE performance, where absolute peak knee flexor force was 13% greater for fast NHE_SSC_ compared to standard slow NHE_ECC_. The fast NHE_SSC_ could therefore be an interesting alternative to the standard slow NHE_ECC_ execution, as it may offer potential advantages for sprint performance, as well as hamstring injury prevention and rehabilitation.

## 1. Introduction

One of the most frequently diagnosed injuries in several sports, including football [1] and track and field [2], is a hamstring strain injury (HSI). According to Kerkhoffs et al. [3], the occurrence rate of HSIs per 1000 h of participation was 0.87 in non-contact sports, such as competitive sprinting, and ranged from 0.92 to 0.96 in contact sports, like football. According to a 21-year study of male professional football conducted by Ekstrand et al. [4], the occurrence of hamstring injuries, along with the total number of days players were absent due to such injuries, doubled during the study period. According to studies [5,6], the risks of re-injury after an acute hamstring injury are between 14 and 63% during a particular season or within two years of the initial injury. Additionally, reinjuries tend to be more severe than the initial HSI [7]. The literature has described at least two distinct types of hamstring strains with differing mechanisms of injury [8]. The first type is associated with high-speed running, while the second type is caused by movements or exercises that stretch the hamstring, such as performing high kicks or executing slide tackles. Recently a study proposed the existence of a third type of hamstring injury, described as “mixed-type”, which involves a combination of both sprinting and stretching-type mechanisms occurring simultaneously [9]. According to the literature, the recovery time for stretching-type hamstring injuries is notably slower compared to sprinting-type hamstring strains [10].

The Nordic hamstring exercise (NHE) is the exercise most included in programs designed to prevent hamstring injuries [11]. A recent meta-analysis and systematic review found that NHE can lower the risk of injuries by 50% among athletes [12], making it one of the most effective strategies for preventing hamstring injuries in sports [13]. It is therefore somewhat paradoxical that the implementation of the NHE remains inadequate in male professional football; however, teams that incorporated NHE into their team training experienced a reduction in hamstring injuries [14]. There are different variations or forms of the NHE that exist [15]. In research, athletes have typically executed the NHE using controlled, slow eccentric muscle action (NHE_ECC_), increasingly leaning their body forward to the furthest extent and then falling to the ground while catching themselves with their hands. However, in sports, trainers and physiotherapists often instruct athletes to perform the NHE in an eccentric–concentric manner, which entails leaning forward during the eccentric phase and subsequently returning to the initial position during the concentric phase [16]. A recent study found that NHE_ECC_ training increases knee flexor fascicle length [17]. The specific adaptations that account for the preventive effects of NHE_ECC_ training on hamstring injuries are still not fully understood. However, it is theoretically possible that elongation of the muscle fascicle may prevent injury by reducing the risk of excessive lengthening [18]. It is worth noting, however, that traditional concentric-eccentric resistance training, which involves the muscle both shortening and lengthening, has likewise demonstrated the ability to promote increases in muscle fascicle length [19,20]. In research on the prevention of hamstring injuries, studies are scarce on standard (NHE_ECC_) compared to eccentric–concentric NHE, in terms of knee flexor force differences. In research, the maximum eccentric force has consistently been observed to be greater than the concentric force [21,22]. However, studies on the force differences between maximal eccentric and eccentric–concentric actions remain limited in the strength training literature [23], and they are scarce in NHE research. Although there is evidence supporting the preventive effect of NHE training [12], various crucial matters, such as determining the appropriate exercise dosage and whether to utilize eccentric or eccentric–concentric muscle actions, remain unclear.

Based on the above argument, Augustsson and Andersson [24] first paid attention to knee flexor force differences between NHE_ECC_ and NHE performed with combined eccentric–concentric muscle action. It was noted that peak knee flexor force for NHE_ECC_ was significantly greater compared to combined eccentric–concentric NHE (5–12%). Furthermore, eccentric–concentric NHE resulted in peak knee flexor force being achieved with significantly less range of forward movement, i.e., at shorter hamstring length. The force–length relationship of the muscle dictates that optimal force is produced at a medium sarcomere length [25] and may at least in part explain why the force generated during eccentric–concentric and NHE_ECC_ was relatively comparative. In the study by Augustsson and Andersson [24], the eccentric-deceleration phase of eccentric–concentric NHE was performed in a slow, controlled manner by the participants. It is well established in the literature that muscle force is increased when a contraction occurs immediately after a prior lengthening action, such as jumping or hopping. This combination of muscle actions described by, e.g., Groeber et al. [26] is known as a stretch-shortening cycle (SSC). Although there are some studies which used higher (initial) movement speed [27,28,29,30], no previous study investigated whether an NHE_SSC_ performed with plyometric and explosive movements using fast muscle action may result in enhanced peak knee flexor force. Furthermore, only one article—a case study involving a single elite athlete—has examined fast NHE_ECC_ [15]. This decelerated version resulted in 20% higher peak moments compared to the standard NHE_ECC_ version. A fast NHE_SSC_ that produces high forces at shorter hamstring length may be an interesting alternative to standard slow velocity NHE_ECC_ at longer muscle length. This could hold true when using NHE to enhance athletic abilities like sprinting [31], but it may also apply to hamstring rehabilitation at which time an athlete may not tolerate a position that involves extended knee angles.

Therefore, the objective of the study was to examine fast NHE_SSC_ vs. standard slow NHE_ECC_. Our hypothesis was that peak knee flexor force would be greater for fast NHE_SSC_ compared to standard slow NHE_ECC_.

## 2. Materials and Methods

### 2.1. Experimental Approach and Trial Design

**Figure 1:**
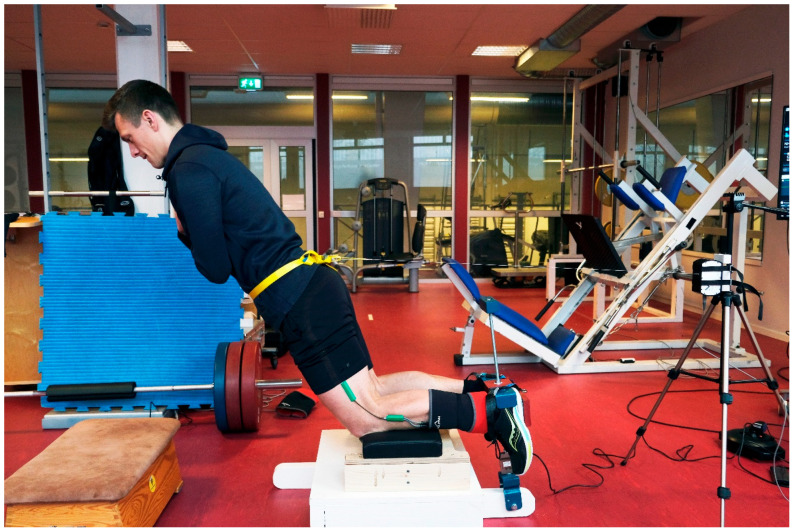
Illustration of the testing set-up, in which the participants performed maximum trials of slow NHE_ECC_ and fast NHE_SSC_ repetitions and where bilateral peak knee flexor force was assessed at the ankle utilizing a load cell. A linear encoder recorded the point at which peak knee flexor force occurred as well as eccentric average velocity during the NHEs. An electronic goniometer was affixed to the outer side of the knee to measure the extent of flexion during the different NHEs.

The study employed a cross-sectional design, where the testing for each participant was conducted within a single test session. The participants were informed that they would perform a rapid NHE_SSC_ before testing, but none of them were familiar with or had experience performing this variation of the exercise, and because of time constraints (upcoming competitions) there was no prior NHE_SSC_ familiarization session. The evaluation of the participants focused on peak knee flexor force differences between fast NHE_SSC_ and standard slow NHE_ECC_. Peak knee flexor force during NHE was assessed at the ankle utilizing a load cell (MuscleLab, Ergotest Technology AS, Langesund, Norway) using a custom device, designed for the purpose of this investigation (see Figure 1). The point at which peak force occurred during the two versions of NHE was recorded by a linear encoder (MuscleLab, Ergotest Technology AS, Langesund, Norway), affixed to the hips of the participants. An electronic goniometer (Biometrics Ltd., Newport, United Kingdom) was affixed laterally to the right knee to measure a range of flexion during the different NHEs.

### 2.2. Participants

Twenty-two elite athletes (competing at both national and international levels) comprising female (*n* = 10) and male (*n* = 7) track and field athletes and male football players (*n* = 5), aged 17–31 years, took part in this research (see Table 1 for the characteristics of participants). One participant, however, was excluded after experiencing calf cramps during testing. The research was conducted during the non-competitive, off-season period of the participants. To be included, the participants had to be highly trained athletes who were familiar with the standard slow NHE_ECC_ and regularly used it in their training. Participants with knee, hip or back injuries within the past six months were excluded from the study. Prior to testing, the participants were informed that they would perform a fast NHE_SSC_; however, none of the participants were acquainted with or had executed this version of the NHE. Prior to their inclusion in the study, all participants received information regarding the risks and benefits associated with their participation. They were given the opportunity to ask questions and clarify any concerns before providing written informed consent.

### 2.3. Procedures

To commence the NHE testing, participants underwent a conventional 5 min warm-up which included hip raises, body-weight squats and standing calf raises. Subsequently, participants, all wearing sports shoes, were positioned in a kneeling stance on the custom NHE device with their arms folded across their chests. The device had shank cushioning (HAM’s HELL, WAW Athletik GmbH, Sandhausen, Germany) and allowed for rigid heel fixation, secured with ankle straps, which is considered an important factor for achieving high-quality execution of NHE [32]. To ensure natural movement of the patella, the shank cushioning was placed beneath the shins, extending up to the tuberosity of the tibia, with the knees positioned at the border, freeing the patella [33]. The participants’ shanks were situated 30 cm above the floor which ensured a sufficient kneeling height to reach full knee extension [32]. Next, a progressive NHE-specific warm-up followed, in which the participants executed three submaximal repetitions of slow NHE_SSC_ at approximately 50% effort. The participants received instructions to execute a gradual and controlled forward lean during the eccentric stage, followed by a return to the initial position during the concentric stage. This was followed by three additional submaximal repetitions of slow NHE_ECC_ performed at around 80% effort. As previously mentioned, none of the participants were acquainted with or had executed the SSC version of the NHE. Therefore, each participant was instructed by the test leader on how to execute the NHE_SSC_ by performing a reversed “trust” exercise in which they were asked to release any hamstring muscle tension before quickly falling forward into the hands of the test leader who stood in front of them. The metaphors “try to fall like a tree” and “to free-fall” [15] were used by the test leader to illustrate the sudden, rapid way the participants preferably would execute the eccentric part of the NHE_SSC_ movement. The familiarization exercise was performed at least three times for each participant, with the test leader allowing the participants to fall forward further and further before catching them each time. The participants were then informed that during maximal NHE_SSC_ testing, they should aim to “free-fall” forward (eccentric phase) just like during familiarization but then quickly decelerate and revert to the initial position (concentric phase). The rest between familiarization and maximal testing was set at 3 min. During this time, the string of a linear encoder (MuscleLab, Ergotest Technology AS, Langesund, Norway), situated at a height of 90 cm on a squat rack positioned behind the participants, was attached to the participants’ hips, at the site of the anterior superior iliac spine, using a strap. The linear encoder recorded the point where bilateral peak knee flexor force occurred for the different NHE variations. An electronic goniometer (Biometrics Ltd., Newport, United Kingdom) was affixed to the outer side of the right knee to measure a range of flexion during the different NHEs. Next, two to three trials of maximal NHE_ECC_ and NHE_SSC_ repetitions, respectively, were conducted, with each trial separated by a 1 min rest period. For the NHE_ECC_, the participants leaned forward and slowly lowered themselves to the ground while maintaining control until the breakpoint and extending their arms out to catch themselves as they approached the ground. During NHE_SSC_, the participants aimed to “free-fall” forward as far as possible and then quickly decelerate (eccentric stage) and revert to the initial position (concentric stage). Bilateral peak knee flexor force during NHE_ECC_ and NHE_SSC_ was assessed at the ankle utilizing a load cell (MuscleLab, Ergotest Technology AS, Langesund, Norway) connected between the rigid heel fixation and the NHE device via a 12 mm threaded rod. The sampling rate for data collection was set at 200 Hz. Moreover, no filter was applied as an analog-to-digital converter for each signal for the load cell and linear encoder, respectively. The sequence of tests administered to the participants was arranged randomly, with half of them beginning with NHE_ECC_ and ending with NHE_SSC_, while the other half started with NHE_SSC_ and concluded with NHE_ECC_. This randomization was accomplished by utilizing the RAND function in Microsoft Excel to produce random numbers in an evenly distributed way. The commands and verbal cues provided to the participants were standardized to ensure consistency throughout testing. All testing and trial performances were overseen by one of the researchers, a sports physical therapist with over 25 years of experience in strength testing and training. To be deemed successful, each repetition of the NHE required the participants to maintain a neutral position of their trunk and hips throughout the entire trial. Data from the load cell were synchronized with that from the linear encoder through the MuscleLab system (V10.21, Ergotest Technology AS, Langesund, Norway).

Alt et al. [32] recently introduced criteria that evaluate the quality of studies on the NHE: Assessing Nordic Hamstring Exercise Quality (ANHEQ). According to the ANHEQ criteria, this study had a “very good” design and reporting quality (10 points of a maximal 13-point score): rigid fixation (2/2), knee position (2/2), kneeling height (1/1), separate familiarization (0/1), diagnostic tools (2/2), feedback of target movement speed (0/2), effects of compromised NHE form (1/1) and documentation of variables related to NHE performance (2/2).

### 2.4. Statistical Analyses

Data analysis was conducted utilizing IBM SPSS Statistics (version 29, IBM, Armonk, NY, USA). To determine the normal distribution of the data, a Shapiro–Wilk test was employed, which indicated that the data met the assumption of normality. As a result, parametric tests were used for significance analysis (*p* > 0.05). The results are presented as mean values accompanied by their respective standard deviations (SDs). Paired samples *t*-tests were employed to detect significant peak knee flexor force differences for fast NHE_SSC_ compared to standard slow NHE_ECC_. Paired samples *t*-tests were utilized to analyze the differences in hip forward distance, measured in cm, attained by the participants at the breakpoint for NHE_ECC_ and at the phase between the eccentric and concentric phases (i.e., the coupling phase) for NHE_SSC_. The difference in eccentric average velocity (m/s) between slow NHE_ECC_ and fast NHE_SSC_ was analyzed using a paired samples *t*-test. The Cohen’s d effect size (ES) was computed to assess peak knee flexor force differences, hip forward displacement attained by the participants and eccentric average velocity between the two types of NHE. The computation involved dividing the difference between the mean values of NHE_ECC_ and NHE_SSC_ by the pooled standard deviations of the different NHE types. According to the established criteria [34], an effect size (ES) of 0.2 was considered small, 0.5 signified a moderate ES and 0.8 denoted a large ES. To investigate the relationship of hip forward distance and eccentric average velocity with peak knee flexor force normalized to body mass, Pearson product-moment correlation coefficients were determined. The strength of the correlation was evaluated according to the following categorization: *r* = 0.00–0.10, indicating a negligible correlation; *r* = 0.10–0.39, indicating a low correlation; *r* = 0.40–0.69, indicating a medium correlation; *r* = 0.70–0.89, indicating a strong correlation and *r* = 0.90–1.00, indicating a very strong correlation [35]. Calculation of sample size: The study’s participant count was established considering a hypothesized 15 to 20% peak knee flexor force difference when comparing NHE_SSC_ and NHE_ECC_ [15,24]. The estimated minimum requirement for participants was 20, ensuring a statistical power of 0.90. The analysis significance levels were defined as *p* < 0.05.

## 3. Results

**Figure 2:**
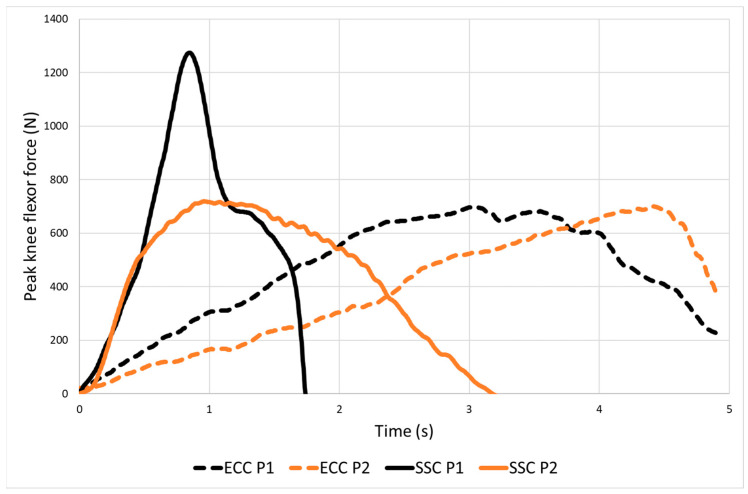
Illustration of the varying capacity of participants to utilize the stretch-shortening cycle (SSC) to enhance muscle performance. Participant 1 (black solid and dotted lines) demonstrated twice as much NHE_SSC_ peak knee flexor force as NHE_ECC_, whereas for participant 2 (orange solid and dotted lines) peak knee flexor force was unchanged between NHE variations.

SSC contributed to an enhanced NHE performance, where bilateral absolute peak knee flexor force was 13% greater for fast NHE_SSC_ vs. standard slow NHE_ECC_ (822 vs. 726 N, *p* < 0.01, ES = 0.54). The hip forward displacement attained by the participants in cm at breakpoint was 32% greater for NHE_ECC_ than at the coupling phase for NHE_SSC_ (54 vs. 41 cm, *p* < 0.001, ES = 1.37). Eccentric average velocity was more than three times higher for NHE_SSC_ compared with NHE_ECC_ (0.38 vs. 0.12 m/s, *p* < 0.001, ES = 3.25). The peak knee flexor force values, the hip forward displacement attained by the participants and the eccentric average velocity for the NHE variations are presented in Table 2. In our study, SSC contributed to an enhanced NHE performance (in terms of peak knee flexor force) for 86% of the participants (18/21). Figure 2 illustrates the varying capacity of participants to utilize the SSC to enhance muscle performance: Participant 1 demonstrated twice as much NHE_SSC_ peak knee flexor force as NHE_ECC_, whereas for participant 2 peak knee flexor force was unchanged between NHE variations.

**Figure 3:**
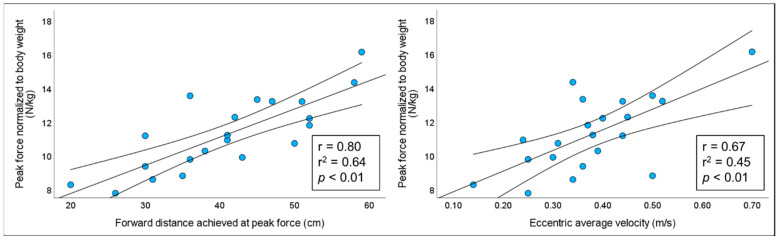
Illustration of correlations between hip forward distance achieved and eccentric average velocity with bilateral normalized peak knee flexor force during NHE_SSC_ in elite athletes *(n* = 21), along with the corresponding 95% confidence intervals.

Strong and moderately strong significant (*p* < 0.01) correlations were noted between hip forward displacement attained by the participants and eccentric average velocity with bilateral normalized NHE_SSC_ peak knee flexor force, *r* = 0.80 and *r* = 0.67, respectively. Figure 3 presents the correlations for hip forward displacement attained by the participants and eccentric average velocity with bilateral normalized NHE_SSC_ peak knee flexor force, respectively.

**Figure 4:**
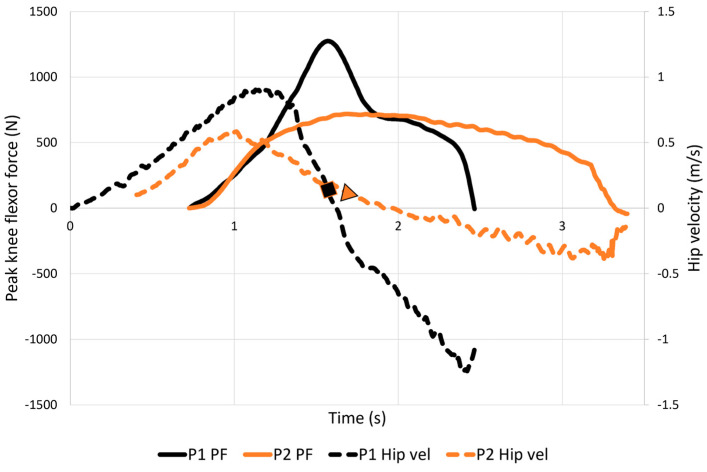
A chart with a primary axis representing peak knee flexor force (PF) and a secondary axis representing hip forward/backward velocity. It illustrates the change of velocity (acceleration and deceleration) during the stretch-shortening cycle (SSC) type NHE and its relation to peak knee flexor force for two of the participants. During the eccentric phase of the NHE_SSC_, large acceleration and deceleration of the body (participant 1, black solid and dotted lines) resulted in higher peak knee flexor force than moderate acceleration and deceleration (participant 2, orange solid and dotted lines). Furthermore, it is noteworthy that peak knee flexor force was attained at the very end of the eccentric deceleration phase at almost zero velocity for both participants (as indicated by the black square and the orange triangle).

Figure 4 shows the change of velocity (acceleration and deceleration) throughout NHE_SSC_ and its relation to peak knee flexor force observed in two participants. Notably, in the eccentric phase of the NHE_SSC_, participant 1 exhibited large acceleration and deceleration, leading to higher peak knee flexor force compared to participant 2, who had more moderate acceleration and deceleration. Moreover, it is evident that both participants achieved peak knee flexor force during the final moments of the eccentric deceleration phase, when velocity approached zero.

Data on knee range of motion were not possible to retrieve for any NHE tests, due to technical problems with the electronic goniometer.

## 4. Discussion

The key findings of this study revealed that SSC contributed to an enhanced NHE performance. Specifically, the absolute peak knee flexor force was 13% greater for fast NHE_SSC_ compared with standard slow NHE_ECC_. Furthermore, it was observed that peak knee flexor force during NHE_SSC_ was achieved with a 32% reduction in the range of movement. Additionally, the eccentric average velocity was more than three times higher for NHE_SSC_ compared with NHE_ECC_.

Therefore, the results verify that a higher eccentric speed of movement has an impact on muscle–tendon stiffness, muscle activation and subsequent force generation during execution [36,37].

To the best of our knowledge, this study represents the first investigation on the effect of a stretch-shortening variant of the NHE compared to the standard slow eccentric execution on peak knee flexor force, amount of forward motion and eccentric average velocity. It was noted that NHE_SSC_ reached higher force values than NHE_ECC_ with less range of movement and higher eccentric average velocity. This finding is consistent with the case study of Alt et al. [15]. The similar extent of peak muscle activity found in this previous study emphasized that the additional strength has been provided by, e.g., the series-elastic structures (tendons). However, to stretch the tendons, both high muscle strength and stiffness are needed [38]. The fast NHE_SSC_ could therefore be an interesting alternative to the standard slow NHE_ECC_ execution, as it may offer potential advantages for sprint performance, as well as hamstring injury prevention and rehabilitation.

Strong and moderately strong correlations were noted between hip forward distance achieved by the participants and eccentric average velocity with NHE_SSC_ peak knee flexor force. In other words, if athletes execute the SSC variation with greater eccentric speed and depth, the peak force will increase. It is likely that there is an ideal combination, a “sweet spot”, of eccentric speed and NHE depth for each athlete, which results in the highest possible force to occur. Also, on the matter of speed, Figure 4 showcases that during the eccentric phase of the NHE_SSC_, large acceleration and deceleration of the body will result in higher peak knee flexor force than moderate acceleration and deceleration. As mentioned in the Methods section, the participants had to be highly trained athletes familiar with the NHE_ECC_ and regularly use this exercise in their training. Before testing, the participants were given information that they would perform a fast NHE_SSC_. However, none of them had prior experience or familiarity with this specific type of NHE exercise. It is therefore particularly intriguing that despite having no prior experience with the NHE_SSC_, 86% (18/21) of the participants still reached higher peak knee flexor force using this variation. Furthermore, during testing, it was quite noticeable that there was room for improvement when it came to executing the NHE_SSC_ in many, if not all, participants.

In a previous study [24], we investigated differences in knee flexor force between NHE_ECC_ and NHE performed with combined eccentric–concentric muscle action, using an NHE test setup using ankle hooks and limited patella glide. Methodologically, it is worth mentioning that our current paper had a superior NHE test setup with improved exercise setup and movement quality (“very good” ANHEQ rating with a total of 10 points) [32], which might have promoted peak force and range of movement. Firstly, the implementation of a shank cushioning freed the patella from any contact forces and thus from any potential strain or stress. Secondly, we used a rigid heel fixation component, further enhancing the quality of movement. This feature ensured that the heel remained securely in place throughout the exercise, allowing participants to generate higher forces compared to fixation by a partner or by a moving hook [33].

Many, if not most, athletes exhibit inadequate strength capacities to sustain high muscle activation during NHE execution at extended knee angles (approximately 30° to 0° knee flexion) [33,39]. However, increased shank inclination [15,40,41] could potentially be employed to enhance the perception of sustained muscle activation and aid in managing the gradually escalating overload induced by gravity during the end range of the NHE [42,43]. Although the reduced range of motion will impair the movement velocity and thus the kinetic energy, the higher specificity of the joint angles might promote this setup.

The present study does have certain limitations. To mitigate the risk of injury, participants performed a warm-up consisting of submaximal NHE repetitions prior to the maximal testing. Nonetheless, we believe that any potential influence of the warm-up on the NHE test results was insignificant. Furthermore, we did not determine the reliability of the two different NHE variations in this study. However, in a prior investigation involving female football players, we assessed the test–retest reliability with the same NHE test setup and noted excellent reliability (as indicated by an intra-class correlation coefficient of 0.95) [16]. Due to time constraints, a prior NHE_SSC_ familiarization session—which probably would have improved NHE_SSC_ test performance—was not conducted. However, it is worth noting that despite the participants’ lack of prior experience with NHE_SSC_, they were able to achieve higher peak knee flexor force with this variation. Furthermore, NHE_ECC_ movement velocity was not controlled in our study. This is due to the fact that we regarded standardized movement velocity redundant for the participants who all were elite athletes with NHE expertise. Lastly, our intention was to examine the knee angle kinematics during the different NHE variations. Unfortunately, due to technical issues with the electronic goniometer, it was not possible to obtain data regarding the knee range of motion for any of the NHE tests.

In perspective, regarding further research on NHE, we propose focusing on a sequential progression of NHE, which involves examining exercise variations such as the NHE_SSC_ and experimenting with, e.g., different shank inclination and hip flexion during NHE. During the late swing phase of sprinting, the length of the muscle–tendon complex is increased. An NHE with flexed hip could be said to mirror the late swing phase of sprinting by lengthening the hamstring muscle–tendon units, which increases the capacity to absorb energy due to the “passive force” [44], provided that the muscles stay highly active [15]. This fact is ensured by the task to complete fast NHE_SSC_ execution, which guarantees a high muscle activity throughout the complete range of motion. This is a major advantage compared to the standard NHE, where muscle activity usually drastically drops at the end of the movement due to insufficient strength capacities at extended knee angles [33]. In conclusion, a stepwise progression of NHE has the potential to benefit athletes with hamstring injuries by facilitating a safer and more effective return to play. Additionally, it offers the opportunity to enhance their athletic prowess, such as improving sprinting and jumping performance [31].

Finally, in relation to hamstring injury prevention, we believe it is worth investigating further in future research whether the fast eccentric–concentric version of the NHE (i.e., the fast NHE_SSC_) is more effective than the traditional slow NHE_ECC_.

## 5. Conclusions

The key findings of this study revealed that SSC contributed to an enhanced NHE performance, with the absolute peak knee flexor force being 13% greater for fast NHE_SSC_ compared with standard slow NHE_ECC_. It was noted that NHE_SSC_ reached higher knee flexor force values than NHE_ECC_ with a less range of movement and higher eccentric average velocity. The fast NHE_SSC_ could therefore be an interesting alternative to the standard slow NHE_ECC_ execution, as it may offer potential advantages for sprint performance, as well as hamstring injury prevention and rehabilitation.

## Figures and Tables

**Table 1:** The participant characteristics for the study (*n* = 22).

Characteristics	*n*	Mean ± SD
Female	10	
Male	12	
Football players	5	
Track and field athletes	17	
Age, year		20 ± 4
Height, cm		178 ± 8
Body mass, kg		72 ± 8
Practice, hours per week		11 ± 3

**Table 2:** Mean and SD bilateral peak knee flexor force values (N), the hip forward distance at peak force (cm) and eccentric average velocity (m/s) by the participants for slow eccentric vs. fast stretch-shortening Nordic hamstring exercise (NHE).

Test Parameters	NHE_ECC_	NHE_SSC_	*p-*Value	Effect Size
Bilateral peak knee flexor force (N)	726 ± 150	822 ± 204	0.008	0.54
Hip forward distance at peak force (cm)	54 ± 9	41 ± 10	0.001	1.37
Eccentric average velocity (m/s)	0.12 ± 0.04	0.38 ± 0.12	0.001	3.25

## Data Availability

The corresponding author can be contacted to request the data presented in this study.
